# Understanding the effect of MXene in a TMO/MXene hybrid catalyst for the oxygen evolution reaction

**DOI:** 10.1038/s41699-023-00377-1

**Published:** 2023-03-10

**Authors:** Daire Tyndall, Lee Gannon, Lucia Hughes, Julian Carolan, Sergio Pinilla, Sonia Jaśkaniec, Dahnan Spurling, Oskar Ronan, Cormac McGuinness, Niall McEvoy, Valeria Nicolosi, Michelle Philippa Browne

**Affiliations:** 1 Centre for Research on Adaptive Nanostructures and Nanodevices (CRANN), Advanced Materials and BioEngineering Research (AMBER) Centre, Trinity College Dublin, Dublin, Ireland; 2 School of Chemistry, Trinity College Dublin, Dublin, Ireland; 3 School of Physics, Trinity College Dublin, Dublin, Ireland; 4 I-Form Research Center, Trinity College Dublin, Dublin, Ireland; 5 Helmholtz-Zentrum Berlin fur Materialien und Energie, 14109 Berlin, Germany

**Keywords:** Materials for energy and catalysis, Materials science

## Abstract

Very recently, it has been reported that mixed transition metal oxide (TMO)/MXene catalysts show improved performance over TMO only catalysts for the oxygen evolution reaction (OER). However, the reasoning behind this observation is unknown. In this work mixed Co(OH)_2_/Ti_3_C_2_T_x_ were prepared and characterized for the OER using ex situ and operando spectroscopy techniques in order to initiate the understanding of why mixed TMO/MXene materials show better performances compared to TMO only catalysts. This work shows that the improved electrocatalysis for the composite material compared to the TMO only catalyst is due to the presence of higher Co oxide oxidation states at lower OER overpotentials for the mixed TMO/MXene catalysts. Furthermore, the presence of the MXene allows for a more mechanically robust film during OER, making the film more stable. Finally, our results show that small amounts of MXene are more advantageous for the OER during long-term stability measurements, which is linked to the formation of TiO_2_. The sensitivity of MXene oxidation ultimately limits TMO/MXene composites under alkaline OER conditions, meaning mass fractions must be carefully considered when designing such a catalyst to minimize the residual TiO_2_ formed during its lifetime.

## Introduction

Electrochemically driven water splitting is a technology under an enormous amount of research in academia and industry in a bid to replace the world’s dependence on fossil fuel-based energy^1,2^. Water splitting can be powered using renewable intermittent energy sources such as wind and solar^3^. During the water splitting process, H_2_O is split into hydrogen and oxygen by applying an external current. The hydrogen can be then used as an energy carrier in a fuel cell to create electricity to power automotive vehicles and infrastructure^4^. Furthermore, if the hydrogen is not needed at that time, it can be stored as a liquid or gas in tanks for later use.

In a water splitting device, i.e., an electrolyzer, hydrogen is produced on the cathode; this reaction is deemed the hydrogen evolution reaction^5^. However, the opposite reaction at the anode, the oxygen evolution reaction (OER), is the most kinetically demanding reaction in water splitting, hence to efficiently produce hydrogen the OER needs to be made more kinetically favorable.

One route to explore to accomplish this, is to design active and stable OER catalysts compared to the current state-of-the-art materials utilized for this oxidative reaction. In alkaline media, the state-of-the-art catalysts for the OER are nickel-based materials^6^. The performance of the nickel catalysts in the anion-exchange membrane (AEM) do not reach that of the IrO_2_/Pt catalysts in a proton-exchange membrane (PEM) device. These materials also exhibit stability issues under intermittent usage which is a major disadvantage when coupled with solar or wind renewable energy for large scale deployment.

First row transition metal oxides (TMOs) are notably one of the most studied classes of materials in the bid to replace the current state-of-the-art OER catalysts. These first row TMOs are also considerably less expensive than current catalysts utilized for the OER. TMOs are an exciting group of materials that possess various intriguing physical properties that can change depending on the oxidation state of the material and are known to be relatively active water splitting catalysts (i.e., they exhibit low OER overpotentials). Furthermore, it has been shown that the shift in the oxidation state of a metal oxide can significantly increase the OER performance of the initial metal oxide under study.

For example, Risch et al. prepared a Co(II) oxide film by electrodeposition of metal salts, and using operando X-ray absorption spectroscopy (XAS) were able to determine that two or more Co(IV) sites are present to make a O–O bond for O_2_ formation in alkaline media^7^. When comparing pure Ni oxide and NiFe oxides, the latter are widely known to be a better OER catalysts. Nocera et al. reported that the superior OER performance of the NiFe oxides is due to the formation of Ni(IV) as a result of the presence of Fe(III)^8^. The Fe(III) promotes the Ni(III)/Ni(IV) redox transaction by increasing the acidity of the aqua/hydroxo OH_x_ groups of the Ni. Hence, this increases the oxy-group presence, which in turn increases the O–O bond formation at lower potentials. The active site of Ni oxide has also been reported to be Ni(IV), i.e., γ-NiOOH, however the pure Ni oxide is a less active material than the NiFe oxide^9^. From these studies, it is logical to state that certain oxidation states of a metal oxide can promote the OER and the OER activity of pure metal oxides can be boosted by the addition of other materials.

As of late, reports on the addition of MXene materials to TMO catalysts for the OER have emerged. MXene materials are derived from MAX (M = transition metal, A = Al or Si, X = C and/or N) phases by various exfoliation and delamination processes^10,11^. MXene materials have many characteristics that coin these materials as “OER compatible”. MXenes are highly conductive, exhibit a high surface area and are hydrophilic, however, they do not contain any active OER metals such as Co or Ni^12^. On the other hand, TMO materials are not very conductive as they are semiconducting by nature. Hence, the addition of MXenes to the TMO in theory could result in an active, conductive, and hydrophilic OER catalyst. The research into these two materials in tandem for the OER is very much in its infancy, however, a TMO/MXene hybrid seems promising.

Various reports have shown that TMO materials in conjunction with the most common MXene, Ti_3_C_2_T_x_, exhibit much improved OER performance than the pure TMO and the pure Ti_3_C_2_T_x_^13–15^^.^ Unfortunately, no operando experiments were conducted to try to understand the reasoning behind the enhanced OER capabilities of the TMO/MXene hybrids. Interestingly though, Lu et al. showed that the ratio of Co_3_O_4_:Ti_3_C_2_T_x_ in the hybrid materials influences the OER. The study by Lu et al. showed that a 10% MXene hybrid outperforms other TMO/MXene hybrids with higher MXene concentrations^13^.

The OER stability of TMO/MXene hybrid catalysts was also investigated by Lu et al. and Yu et al.^13,14^. Lu et al. multi-cycled their Co_3_O_4_:Ti_3_C_2_T_x_ catalyst for 2000 cycles in the OER region. The Co_3_O_4_:Ti_3_C_2_T_x_ lost a significant amount of activity (~1000 mV) over the 2000 cycles^13^. Furthermore, Yu et al. conducted chronopotentiometry for 60 h of a FeNi-LDH/Ti_3_C_2_T_x_ material on Ni foam and showed that the potential required to reach a current density of 10 mA cm^−2^ decreased by ~500 mV in the 60-h testing window^14^. Hence, to make these hybrid materials more stable, a more thorough investigation of these hybrid materials during the OER needs to be carried out.

In this work, we will try to understand why these TMO/MXene hybrid materials exhibit better OER activity compared to their pure counterparts. To investigate the OER activity of the hybrid materials, operando Raman spectroscopy will be utilized. The effect of the ratio of the TMO/MXene will also be explored, and in conjunction with various material characterization techniques we will elucidate how the ratio influences the OER activity of the active material.

## Results and discussion

### Synthesis and characterization of the electrocatalysts

In this work, the aim is to understand the reasoning behind the already known enhancement that the TMO/MXene hybrid exhibits. The MXene material will be Ti_3_C_2_T_x_ as this is the only MXene investigated to date in these hybrids for the OER. While the TMO component will be Co hydroxide as it is a known active OER material.

Before the composite was prepared, the pure Co oxide was synthesized by a low temperature precipitation method and will be denoted as “pure Co” from here on in. Briefly, cobalt nitrate, NaCl, hexamethyltetramine and H_2_O/EtOH were added to a reaction vessel and heated at 90 °C for 1 h. Additionally Ti_3_C_2_T_x_, the MXene component, was prepared by the “mild” HCl and LiF MXene synthesis route^16^. In this work the hybrid TMO/MXene composite was fabricated by mechanically mixing pure Co oxide and Ti_3_C_2_T_x_ in a H2O/EtOH solution. The composite was made with 99% vol of the pure Co oxide and 1% vol of the Ti_3_C_2_T_x_. This sample will be denoted as “1% MXene” from here on in.

**Fig. 1:**
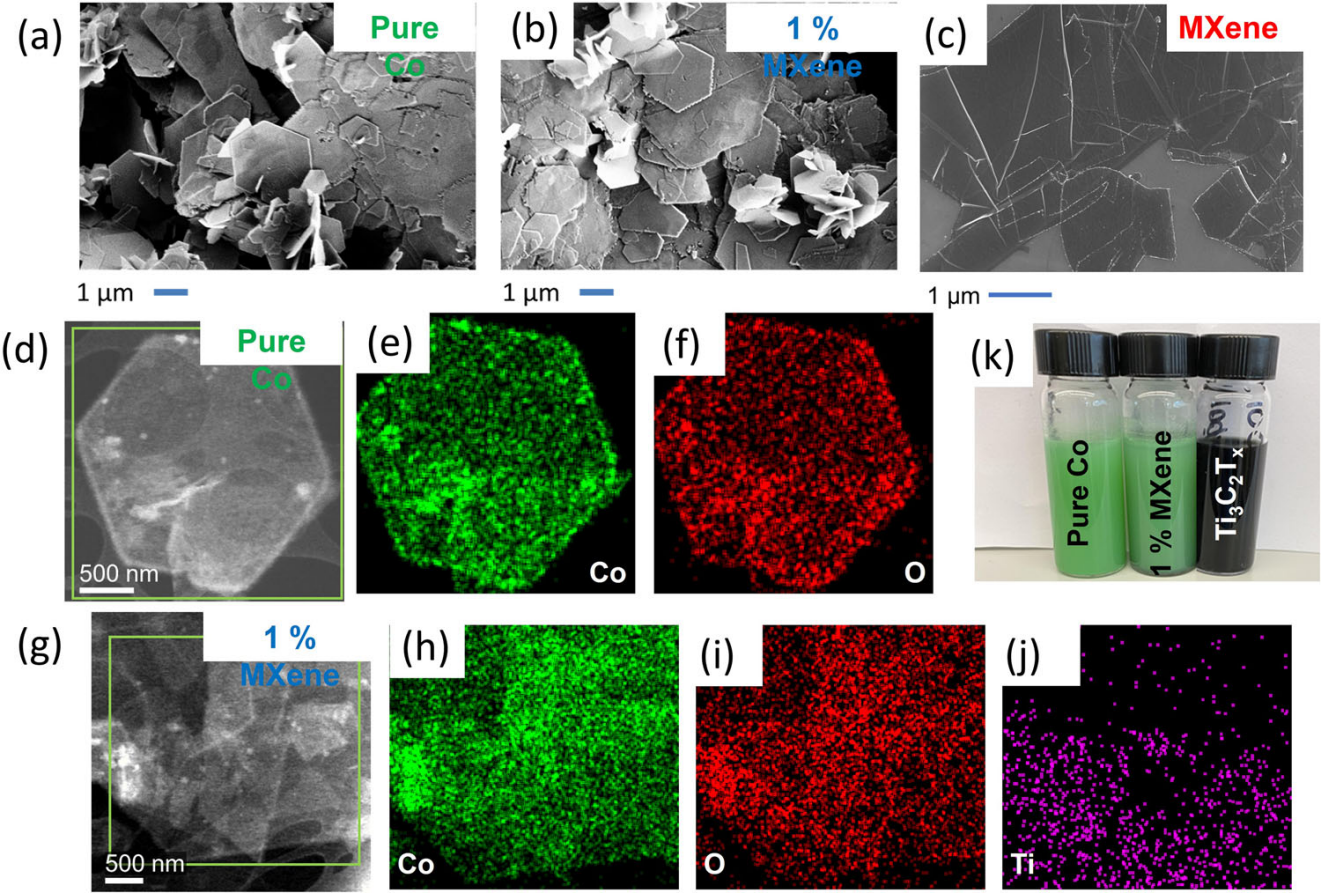
Morphological characterization. Scanning electron microscopy images of (**a**) Pure Co, (**b**) 1% MXene, (**c**) pure MXene. **d** Scanning transmission electron microscopy image of the pure Co. **e** Energy dispersive X-ray map of the Co Kα corresponding to **d**. **f** Energy dispersive X-ray map of the O Kα corresponding to **d**. **g** Scanning transmission electron microscopy image of the 1% MXene. **h** Energy dispersive X-ray map of the Co Kα corresponding to **g**. **i** Energy dispersive X-ray map of the O Kα corresponding to **g**. **j** Energy dispersive X-ray map of the Ti Kα corresponding to **g**. **k** Photo of the pure Co, 1% MXene and the pure MXene/Ti_3_C_2_T_x_ ink dispersions.

The morphology of all three materials: the pure Co, the 1% MXene and the pure MXene was investigated using scanning electron microscopy (SEM). The pure Co and the 1% MXene materials both revealed particles consisting of different sized full and fragmentated hexagonal sheets (Fig. 1a, b) respectively (confirmed by transmission electron microscopy (TEM), Supplementary Fig. 1), while the SEM image of the pure MXene reveals a sheet-like morphology (Fig. 1c).

From the SEM images, it is not evident that the Ti_3_C_2_T_x_ is present in the 1% MXene composite. Hence, energy dispersive X-ray (EDX) spectroscopy was performed to determine the presence of the Ti_3_C_2_T_x_ in the 1% MXene sample. The scanning transmission electron microscopy (STEM) image of the pure Co can be observed in Fig. 1d with the corresponding EDX maps clearly showing the distribution of cobalt (Fig. 1e) and oxygen throughout the sample. When comparing the STEM image and the corresponding EDX maps of the pure Co (Fig. 1d–f), to the STEM image/EDX maps of the 1% MXene (Fig. 1g–j), the presence of Ti from the Ti_3_C_2_T_x_ clearly visible in the sample, even though the 1% MXene is the same color as the pure Co sample in Fig. 1k. Furthermore, the STEM image and EDX maps of the MXene (Supplementary Fig. 2) confirm the presence of Ti and C in the Ti_3_C_2_T_x_ material. Furthermore, no oxygen is detected from the corresponding EDX spectrum of the pure MXene sample (Supplementary Fig. 2d).

The determination of the chemical nature of OER materials before, during and after operation is extremely important in order to design/improve the catalytic activity of materials for this reaction. Hence before OER, the chemical nature of the as-prepared pure and hybrid materials was investigated using a combination of X-ray photoelectron spectroscopy (XPS) and Raman spectroscopy.

**Fig. 2:**
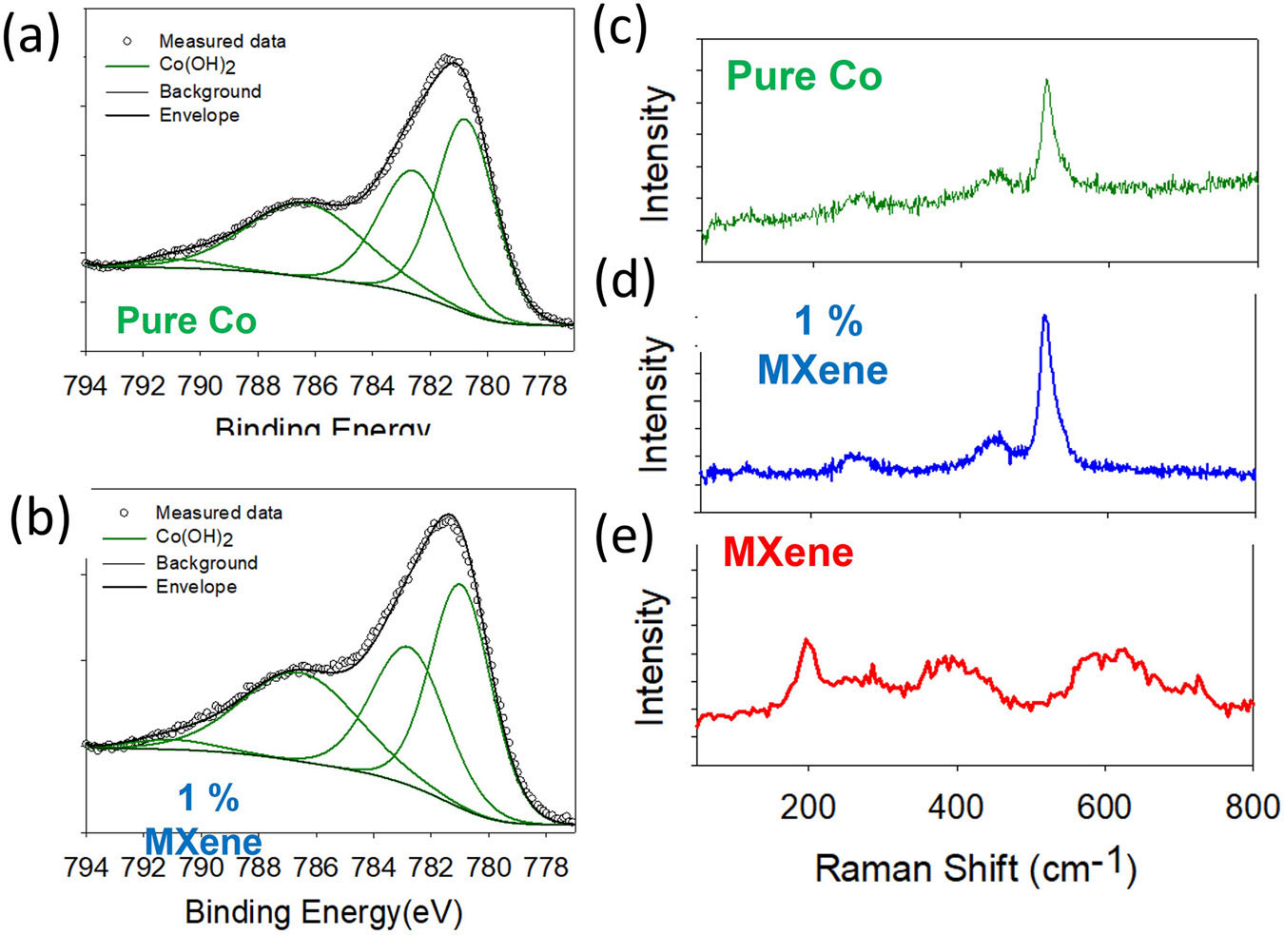
Structural characterization. X-ray photoelectron spectroscopy high resolution Co2*p*3/2 core level for (**a**) pure Co, (**b**) 1% MXene. Raman spectra (using a 532 nm laser) of (**c**) pure Co, (**d**) 1% MXene and (**e**) pure MXene/Ti3C2Tx.

From the XPS analysis, the oxidation state of the cobalt component in the pure Co and 1% MXene materials was investigated by fitting the Co2_*p*3/2_ core level (Fig. 2a, b) respectively. The high resolution Co2_*p*3/2_ core level pure Co and 1% MXene materials were fitted to a Co^2+^ multiset with no contributions related to higher Co oxidation states i.e., Co^3+^ or Co^[4+ 17,18^. This result shows that the Co oxidation state of the layered Co(OH)_2_ flakes were not altered due to physical manipulation during the preparation of the hybrid 1% MXene material.

Raman spectroscopy was also carried out to determine the chemical structure of the three materials beyond the surface by assigning the active modes detected in Fig. 2c–e. The two most intense Raman modes in the pure Co and the 1% MXene samples are present at ~520 and 450 cm^−1^ (Fig. 2c, d) respectively. The peak at ~520 cm^−1^ can be assigned to the Co–O (Ag) symmetric stretching mode in the Co(OH)_2_ lattice^19^. While the Raman mode at ~450 cm^−1^ can be attributed to the O–Co–O bending mode in the Co(OH)_2_ lattice^19^. Finally, to determine the chemical structure of the MXene and to further confirm the material was not oxidized before any electrochemical measurements, the Raman peaks of the Ti_3_C_2_T_x_ sample were investigated. From Fig. 2e, the Raman modes present are indicative of those present in a Ti_3_C_2_T_x_ flake sample probed using a green laser^20^. The peaks at ~200 and 725 cm^−1^ can be attributed to the A_1g_ (C) and the A_1g_ (Ti, O, C) out of plane modes, respectively. While the broad peaks between 345–470 cm^−1^ can be assigned to in-plane (*E*_*g*_) vibrations of the surface terminations bonded to Ti and the peaks at 560–680 cm^−1^ can be attributed to carbon in and out of plane vibrations^20^. Hence, the Raman analysis shows that the pure MXene sample (Ti_3_C_2_T_x_) is not oxidized, which is further confirmed by no O Kα signal in the STEM-EDX map (Supplementary Fig. 2). Interestingly, the Raman also suggests that there is no evidence of any Ti_3_C_2_T_x_ in the 1% MXene sample (Fig. 2d). However, from the STEM-EDX map of the 1% MXene (Fig. 1j), it is evident that the Ti_3_C_2_T_x_ is presence from the presence of the Ti Kα map.

#### Initial oxygen evolution reaction studies

**Fig. 3:**
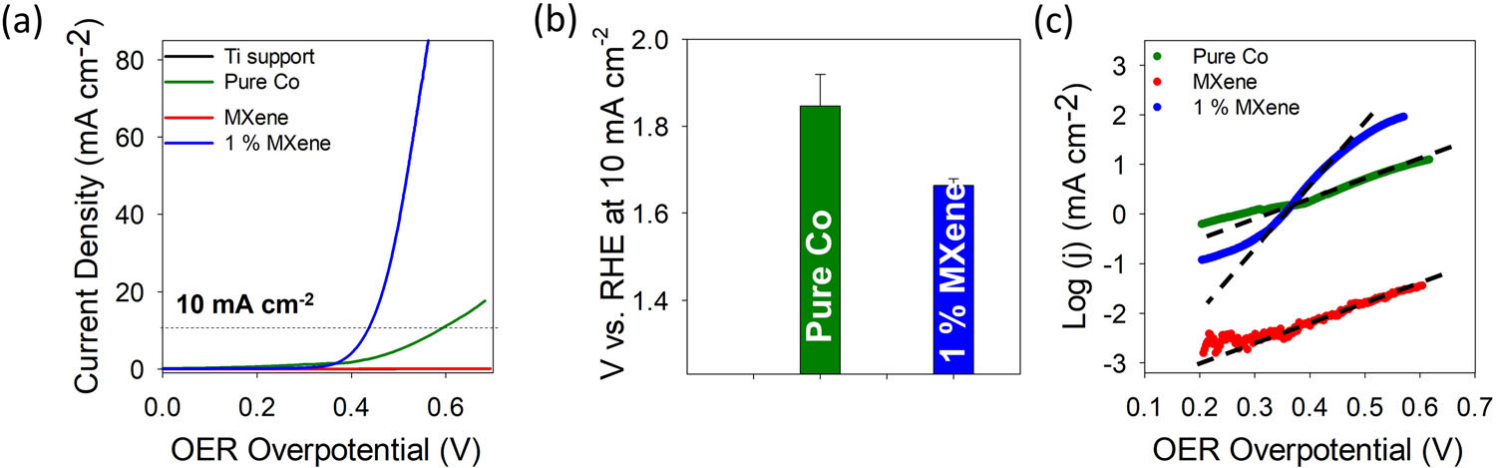
Initial oxygen evolution studies. **a** Linear sweep voltammetry curves at a scan rate of 1 mV dec^−1^ at a rotation speed of 1600 rpm for the Ti support, pure Co, 1% MXene and the pure MXene materials. **b** Bar chart showing the potential (V vs. RHE) need to reach a current density of 10 mA cm^−2^ for the pure Co and 1% MXene. Standard deviations calculated from three different freshly prepared electrodes for each material. **c** Tafel plots for the pure Co, 1% MXene and the pure MXene materials. Dash lines indicate slope for each plot.

To carry out the OER measurements on the three samples characterized in Figs. 1 and 2, ink suspensions containing each material were prepared and drop cast onto a Ti RDE support, more information in the experimental section. The Ti RDE support has no OER activity therefore does not alter/enhance any materials drop cast on it toward the OER (Supplementary Fig. 3). The initial inherent OER catalytic activity of the pure and hybrid materials were carried out by linear sweep voltammetry (LSV) measurements (Fig. 3a).

From the LSV curves it is evident that the hybrid 1% MXene material exhibits improved OER capabilities when compared to the two pure materials; pure Co and MXene, as the LSV curve for the 1% MXene is shifted to more cathodic potentials and reaches higher current densities over the same potential window. Furthermore, the potential at which the 1% MXene and the pure Co materials reach a current density of 10 mA cm^−2^ is 1.66 V ± 0.01 vs. reversible hydrogen electrode (RHE) and 1.84 V ± 0.07 vs. RHE, respectively (Fig. 3b). From Fig. 3a, the Ti_3_C_2_T_x_ material can be deemed quite inactive for the OER. Interestingly, for this particular combination/ratio of materials for the OER (i.e., 1% MXene with Co(OH)_2_ LDHs), the physical mixing of the two components results in better OER activity when compared to the two components being chemically functionalized in a one pot synthesis (Supplementary Fig. 4).

**Fig. 4:**
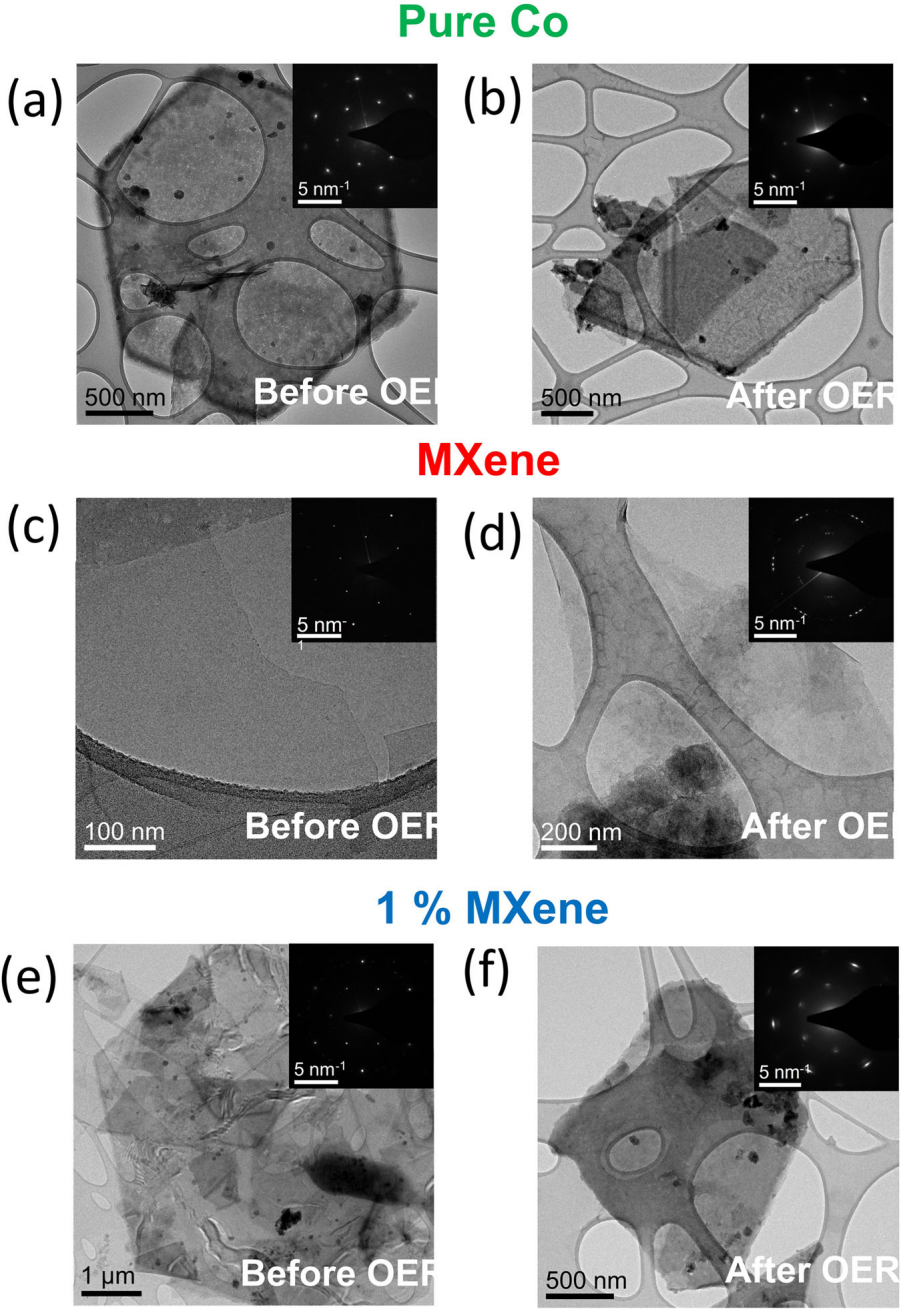
TEM investigation before and after OER. TEM images and corresponding SAED patterns for the pure Co (**a**) before OER, (**b**) after OER. TEM images and corresponding SAED patterns for the pure MXene (**c**) before OER, (**d**) after OER. TEM images and corresponding SAED patterns for the 1% MXene (**e**) before OER, (**f**) after OER.

**Fig. 5:**
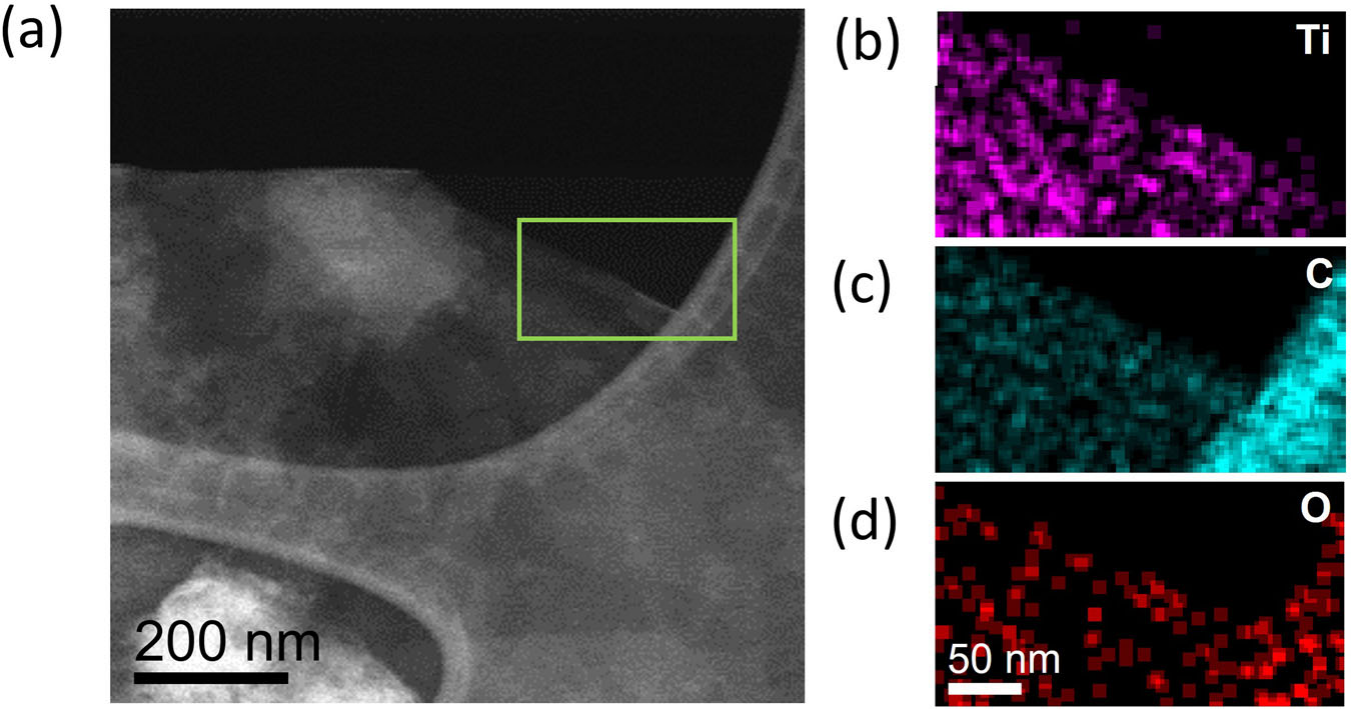
STEM-EDX maps of the MXene material post OER. **a** STEM image, **b** corresponding Ti Kα map, **c** corresponding C Kα map and **d** corresponding O Kα map.

**Fig. 6:**
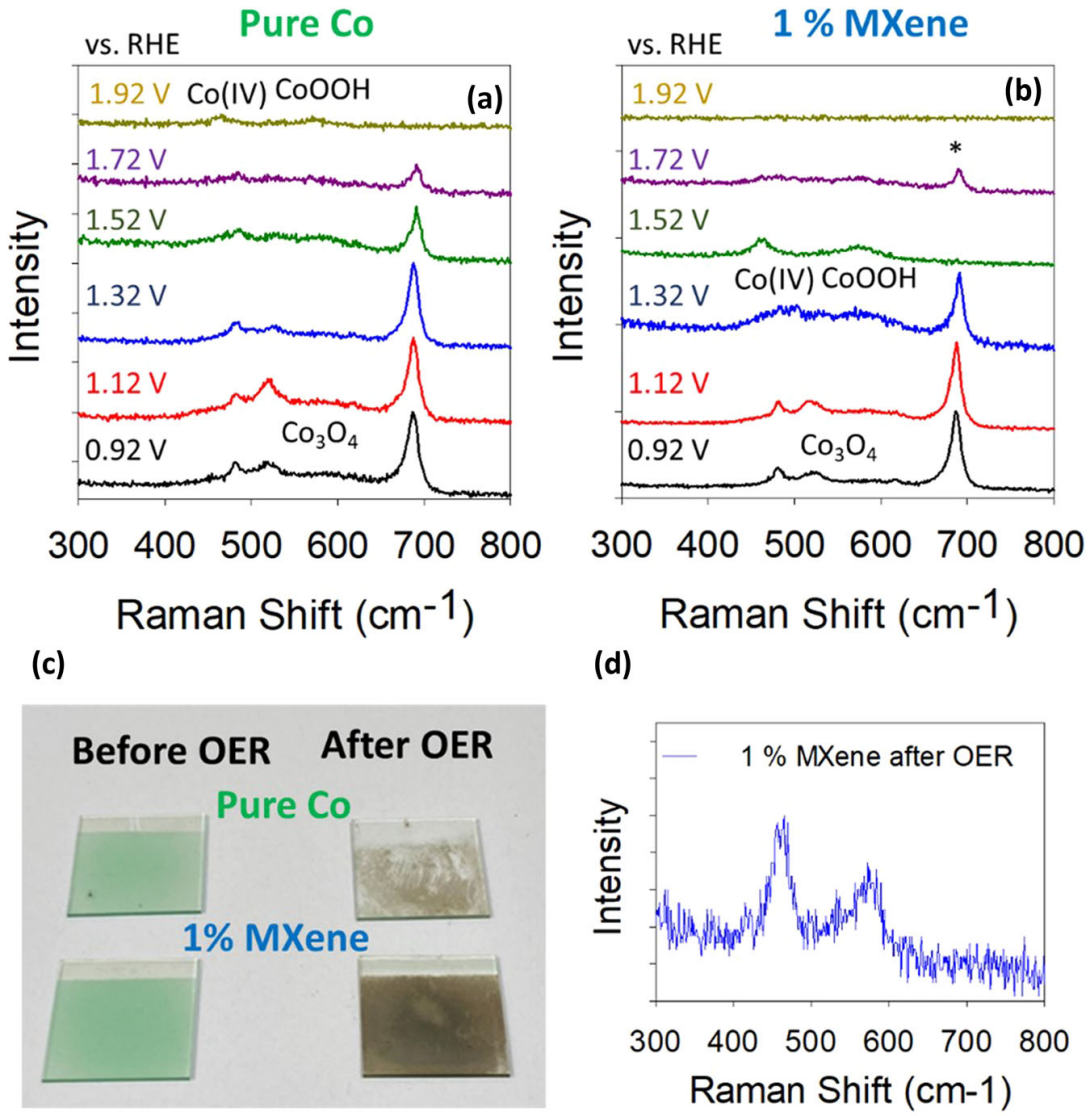
OER active site investigation. **a** Raman spectra for the pure Co catalyst from the potentials of 0.92–1.92 V vs. RHE. **b** Raman spectra for the 1% MXene catalyst from the potentials of 0.92–1.92 V vs. RHE and **c** Photo of the pure Co and 1% MXene catalysts before and after OER. **d** In situ Raman spectrum of the 1% MXene in cell after OER when the applied potential was powered off.

Additionally, the Tafel slope values were also determined to give insight into the kinetics and the efficiency of the materials under study (Fig. 3c). In this case, the lower the Tafel slope value the faster the material is at producing O_2_^21^. The Tafel slope values of the pure Co, 1% MXene and Ti_3_C_2_T_x_ materials are 250, 73 and 260 mV dec^−1^, respectively. The lower Tafel slope value of the 1% MXene compared to the two pure materials confirms the better OER performance of this material. To elucidate the possible reason behind the 1% MXene exhibiting superior initial OER activity when compared to the pure Co(OH)_2_ and MXene materials, all three materials were compared for any changes in the morphology and structure before and after OER using STEM analysis (Figs. 4 and 5), while the pure Co and 1% MXene were investigated by operando Raman spectroscopy for the change in structure during operation (Fig. 6).

To investigate the morphology and crystal structure of the pure and composite materials, post OER TEM imaging and selected area electron diffraction (SAED) were conducted. From the TEM image in Fig. 4a, the as-synthesized pure Co material has a sheet-like morphology with the SAED spots corresponding to (101̅0) and (011̅0) planes of crystalline Co(OH)_2_. For the pure Co material after OER (Fig. 4b), the TEM image shows that the morphology is once again sheet-like with a crystalline SAED pattern exhibiting spots corresponding this time to (101̅0) and (011̅0) planes of CoOOH. For the pure MXene, from the TEM images before and after the OER (Fig. 4c, d) respectively, the sheet-like morphology stayed intact post OER. Furthermore, the crystal structure of the MXene before and after OER was also unchanged as the corresponding SAED patterns before and after OER exhibited spots corresponding to (101̅0) and (011̅0) planes of Ti_3_C_2_. Even though the morphology and the crystal structure remained constant for the MXene materials post OER from the TEM imaging and SAED patterns, there was also a O Kα signal detected in the EDX maps after OER alongside the Ti and C Kα maps (Fig. 5).

Similar to the pure Co and pure MXene samples, the 1% MXene exhibited a sheet-like morphology from the TEM images (Fig. 4e). The corresponding SAED patterns can be indexed to the (101̅0) and (011̅0) planes of Co(OH)_2_. For the 1% MXene after OER (Fig. 4f), the TEM image and corresponding SAED pattern shows spots corresponding to the (101̅0) and (011̅0) planes of CoOOH with minor spots indexed to the Co(OH)_2_ structure. Unlike the MXene sample before OER, no Ti_3_C_2_ or oxidative species of Ti_3_C_2_ were detected using SAED for the 1% MXene. This is possibly due to the low volume of Ti_3_C_2_T_x_ in the sample.

The operando Raman measurements were carried out by acquiring Raman spectra at various potentials before and after the thermodynamic potential for OER (i.e., 1.23 V vs. RHE) in an attempt to understand the nature of the Co active sites present in the pure Co and 1% MXene materials during the evolution of O_2_. In this study, the determination of the active sites for the pure Co is critical to understand the active sites in the 1% MXene material. For the pure Co material at potential pre-OER (i.e., 0.92–1.12 V vs. RHE), the Raman spectra correspond to that of Co_3_O_4_ (Fig. 6b)^22^. During the OER from the potential of 1.32–1.72 V vs. RHE, Co_3_O_4_ is still the dominant Co oxide, however the intensity of the peak at ~688 cm^−1^ decreases as the potential increases which could suggest the depletion of Co_3_O_4_ in the catalyst layer. Interestingly, at a high OER potential of 1.92 V vs. RHE, the Raman spectrum has changed. At this potential the peak at ~688 cm^−1^ assigned to the Co_3_O_4_ has disappeared, while two new Raman peaks at 462 and 576 cm^−1^ are now present, indicating that CoOOH/Co(III) is now present on the electrode^22^. Furthermore, the peak at 576 cm^−1^ can be assigned to Co(IV)/CoOx as reported by Bell et al.^23^.

On the other hand, for the 1% MXene, Co_3_O_4_ is present at the potentials prior to the OER (i.e., 0.92–1.12 V vs. RHE). However, after the onset of the OER, the presence of the various Co oxide species at the different potentials significantly differs to the pure Co (Fig. 6b). At a potential of 1.32 V vs. RHE, the formation of a CoO_x_ peak at 576 cm^−1^ is already evident, alongside the initial Co_3_O_4_. Then at a significantly lower potential (1.52 V vs. RHE) compared to the pure Co (1.92 V vs. RHE), the two defined peaks at 462 and 576 cm^−1^, with the latter indicating higher Co oxides are present. Furthermore, the *A*_1g_ peak of the Co_3_O_4_ at a potential of 1.72 V vs. RHE (indicated by the * in Fig. 6b) is detected alongside the CoOOH and CoO_x_ peaks at a potential of 1.72 V, this is more visible in the Raman spectra in Supplementary Fig. 5, which may indicate that not all of the Co_3_O_4_ on the electrode oxidizes to higher oxidation states at one time. Due to an extensive amount of O_2_ bubbles at 1.92 V vs. RHE for the 1% MXene, a Raman spectrum was unable to be acquired.

### OER mechanism discussion for 1% MXene

When comparing the operando Raman spectra and the LSV curves (Fig. 3) of the pure Co and 1% MXene materials, it is very plausible that the superior OER activity of the 1% MXene is due to the presence of the higher oxidation state CoO_x_ species at lower OER overpotentials than for pure Co. The presence of the MXenes in the hybrid 1% MXene catalyst reduces the overpotential at which Co(IV) species are formed thus improving the catalytic performance and the evolution of O_2_. This explanation for the improved OER performance of the 1% MXene materials over the pure Co(OH)_2_ is coherent with other reports in the literature, which indicate that Co(IV)/CoOOH species are needed for O_2_ evolution at Co based OER catalyst^7,24,25^. Furthermore, from the Raman spectra (Fig. 6d) and the post OER SAED pattern of the 1% MXene material (Fig. 4f), it is plausible to state the Co oxide material remaining after catalytic operation is crystalline CoOOH and not Co_3_O_4_.

Another reason behind the 1% MXene film exhibiting better initial OER capabilities could be the mechanical stability of the film during the OER. In Fig. 6c, the pure Co and the 1% MXene films on an ITO support before and after OER can be observed. Before OER, both films are homogenously sprayed over the ITO glass and are both the same shade of green. After the OER, both films turned to brown indicating the oxidation of the initial green Co(OH)_2_ to higher Co oxides which is confirmed by Raman spectroscopy and SAED. However, interestingly, the film of the Co(OH)_2_ has physically degraded, while the 1% MXene film is still intact. Therefore, the addition of only 1% volume of MXene not only increases the initial OER capabilities of the Co(OH)_2_, it also improves the physical properties of the film during OER.

#### OER stability: effect of MXene % volume in hybrid material

**Fig. 7:**
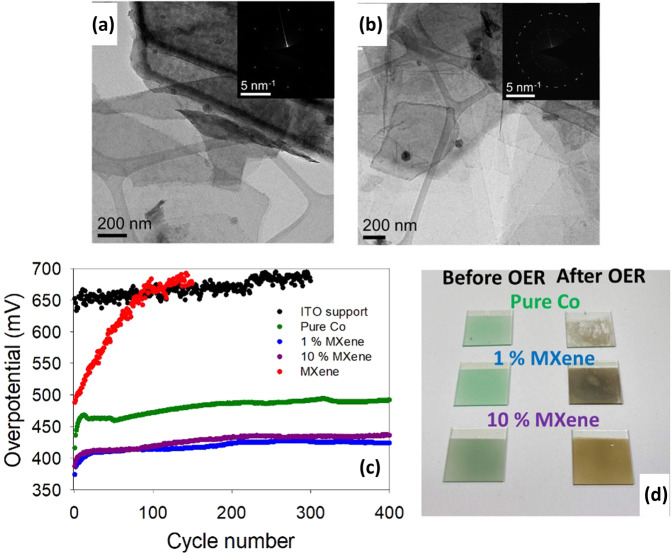
OER stability study. **a**, **b** TEM images and corresponding SAED patterns for the 10% MXene before OER. **c** Effect of multi-cycling into and out of the OER region for the ITO support, pure Co, 1% MXene, 10% MXene and the pure MXene materials. **d** Photo of the pure Co, 1% MXene and 10% MXene catalysts before and after OER.

To study the effect MXene has on intermittent OER stability, four materials were prepared which included the pure Co, 1% MXene and the pure MXene from the initial OER measurements. Additionally, a 10% MXene catalyst was prepared in the same manner as the 1% MXene, see experimental details for more information. From Fig. 7a, b, the TEM images and corresponding SAED patterns show the (101̅0) and (011̅0) planes of Co(OH)_2_ and the (101̅0) and (011̅0) planes of Ti_3_C_2_, respectively. The 10% MXene catalyst was synthesized to understand the role the MXene component plays on OER stability as MXenes are known to be inherently less stable during oxidative processes but clearly from Fig. 3 increase the OER activity of metal oxides alone^26^.

For alkaline water electrolysis, the effect that the continuous “shut down/powering on” of the cell has on the OER material is of critical importance. This is because alkaline water electrolyzers are used intermittently i.e., not on a continuous basis. In a lab setting, this “shut down/powering on” procedure can be mimicked by continuously sweeping the potential into/out of the OER region and plotting the overpotential at a current density in the OER to monitor any changes. Figure 7a shows the plot of the cycle number against the overpotential at a current density of 1 mA cm^−2^ to the pure Co, 1% MXene, 10% MXene, pure MXene and the bare ITO support.

The ITO supports were used for the stability studies as the materials were able to be scaled up from an active area of 0.071 cm^−2^ (rotating disc electrode (RDE) set-up from the initial OER performance study) to 6.25 cm^−2^. Furthermore, the materials were sprayed onto the ITO supports which is the technique used for the preparation of membrane electrode assemblies for electrolyzer devices. The ITO shows very poor OER activity during continuous multi-cycling in the OER region. The pure MXene catalyst initially exhibits an overpotential of ~490 mV at a current density of 1 mA cm^−1^; however, it rapidly loses its OER activity with increasing cycle number, which could be correlated to the increase in oxygen species in the STEM-EDX maps for this material after OER (Fig. 5).

The pure Co, 1% MXene and the 10% MXene all seem relatively stable during multi-cycling. Furthermore, the multi-cycling stability tests again confirm that the 1% MXene exhibits better OER activity. In relation to the 10% MXene, the material exhibits similar initial overpotentials compared to the 1% MXene. However, as the cycle number increases, the 10% MXene catalyst needs a higher overpotential to reach 1 mA cm^−2^. From Fig. 7d, the color of the 10% MXene before OER is darker than the 1% MXene, showing that the 10% MXene indeed does have a higher MXene content. However, after OER, the 10% MXene film is a lighter shade of brown than the 1% MXene due to the larger amounts of the TiO_2_; which is a shade of white. This color change alone may be a strong indication of an oxidation mechanism for MXenes which can act to degrade the composite catalyst in high percentages. This point will be clarified in the following characterization study of the 10% MXene material.

### MXene degradation influence on OER

**Fig. 8:**
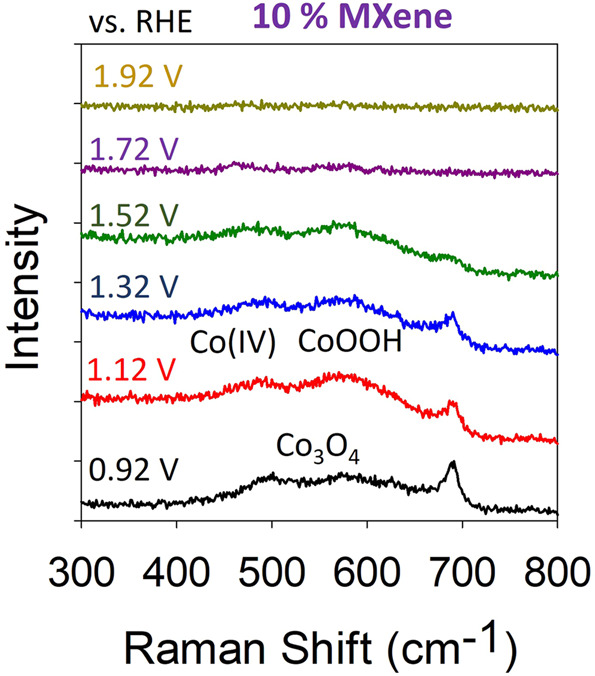
Further OER active site investigation. Raman spectra for the 10% Raman catalyst from the potentials of 0.92–1.92 V vs. RHE.

The loss in the OER activity over time for the 10% MXene compared to the 1% MXene can be explained by a combination of comparing the color of the catalyst films before/after OER alongside operando Raman measurements, post-mortem TEM-SAED and post-mortem XPS. As mentioned, the initial OER activity of the 10% MXene is better when compared to the 1% MXene, however, the stability for the 10% MXene decreases over time. To investigate why the 10% MXene is better for the OER initially, operando Raman spectroscopy was conducted (Fig. 8).

From the operando Raman measurements, it is evident that the higher Co oxidation states are active in the 10% MXene materials at even lower potential when compared to the 1% MXene (Fig. 6b). The presence of these higher oxidation states clearly promotes the OER even earlier for the 10% MXene. Not only does this result show why the 10% exhibits better initial OER performances but it also shows that the addition of more MXene to Co oxide improves the performance.

**Fig. 9:**
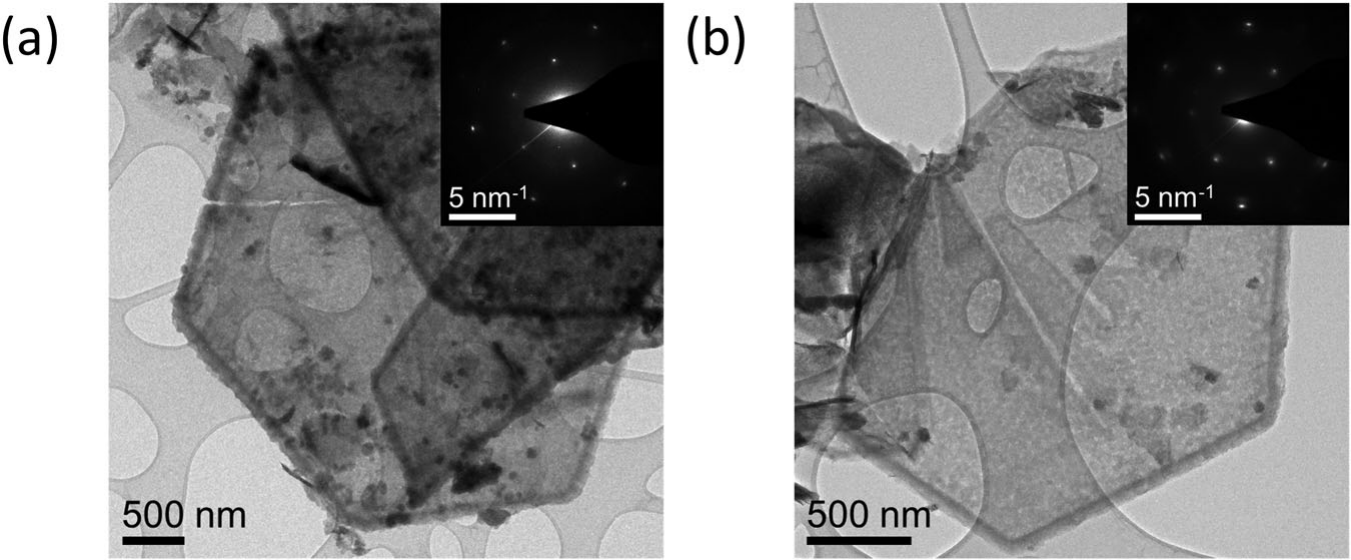
Morphological and structural changes of the 10% MXene before and after OER. TEM images and corresponding SAED patterns for the 10% MXene after OER **a** Area 1 and **b** Area 2.

The TEM imaging and SAED also confirm the oxidation of the Co(OH)_2_ to higher oxidation states for 10% MXene. In relation to the Co material after OER for this sample (Fig. 9a), the TEM image and corresponding SAED pattern show spots corresponding to the (101̅0) and (011̅0) planes of CoOOH. The same can be seen in Fig. 9b, but with minor spots indexed to the (101̅0) and (011̅0) planes of the original Co(OH)_2_ structure. Hence, after OER a slight amount of Co(OH)_2_ is still present which is likely due to the electrolyte not reaching this material and hence not oxidizing. This is similar to what is observed after the OER for the 1% MXene. TEM images and corresponding SAED patterns were recorded at various locations on the 10% MXene sample, but no Ti_3_C_2_ or TiO_2_ was detected after OER to determine any degradation processes occurring for the 10% MXene (Fig. 9a, b).

**Fig. 10:**
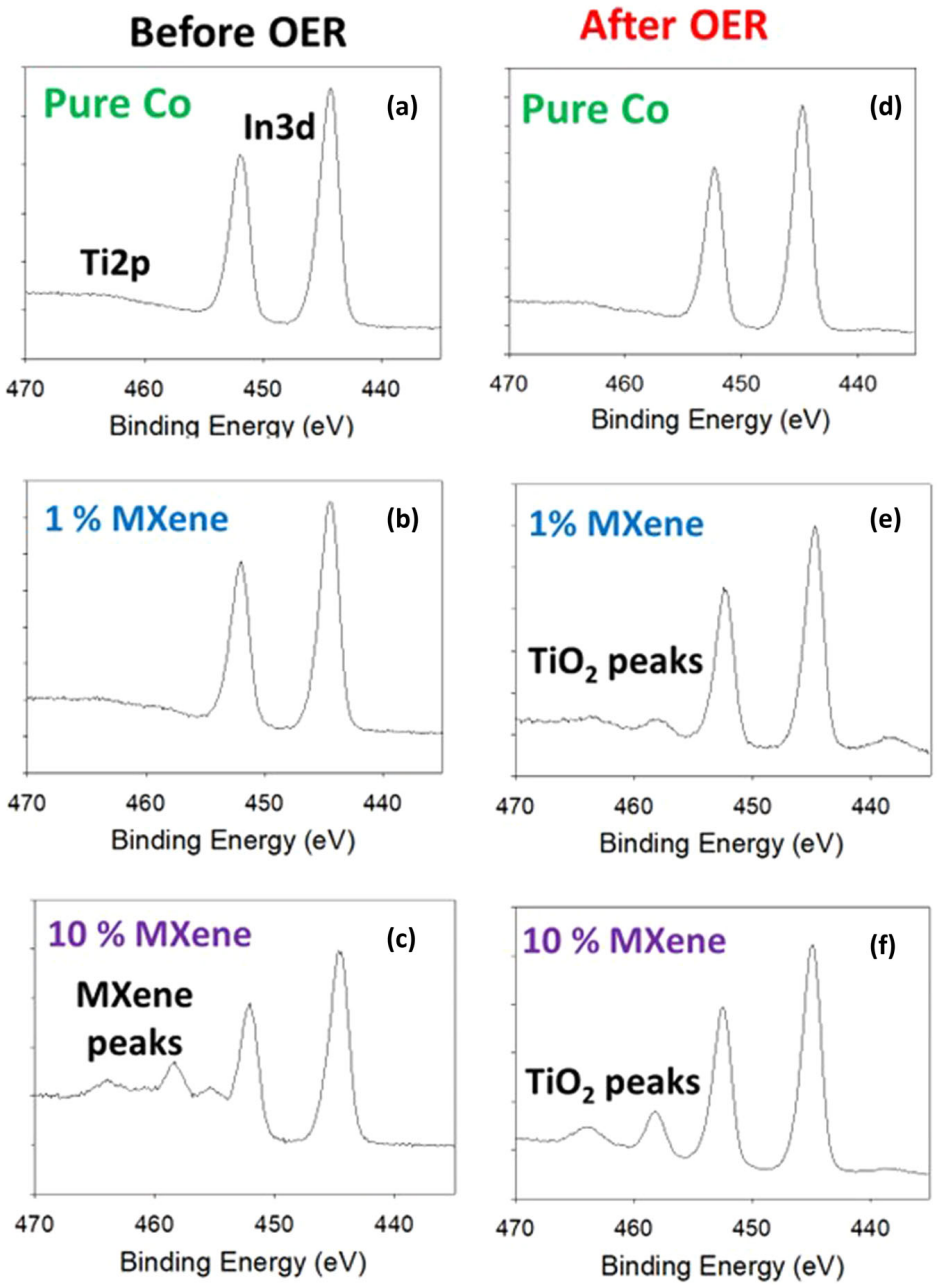
Investigation into structural changes before and after OER. X-ray photoelectron spectroscopy high resolution Ti2*p*/In3*d* core levels before OER for (**a**) pure Co, (**b**) 1% MXene, (**C**) 10% MXene. X-ray photoelectron spectroscopy high resolution Ti2*p*/In3*d* core levels after OER for (**d**) pure Co, (**e**) 1% MXene, (**F**) 10% MXene.

To get a more representative picture of the 10% MXene material, XPS was utilized. XPS can detect TiO_2_ and Ti_3_C_2_ quite effectively and over a wider sample area which allows a more representative overview of a material compared to SAED. As MXenes are known to degrade under oxidative potentials, XPS analysis of the high resolution Ti2*p* core level region was undertaken on the pure Co, 1% MXene and 10% MXene to detect any degradation of the MXene component in the hybrid materials. The In3*d* core level is present in all of the XPS spectra as the In signal measured is from the ITO supports (Fig. 10).

Clearly for the pure Co, no titanium species were detected before OER (Fig. 10a). There was also no titanium species detected in the 1% MXene catalysts (Fig. 10b), which correlates with the SAED measurements (Fig. 4e, f). For the 10% MXene, there were clear MXene peaks detected before OER (Fig. 10c)^27^.

Then after OER, as expected, again no Ti species were detected for the pure Co species (Fig. 10d). However, for the 1% and 10% MXene TiO_2_ was present on the surface of the catalyst (Fig. 10e, f). This is an interesting result regarding the 1% MXene, as the XPS analysis of the 1% MXene before OER shows no signs of Ti_3_C_2_T_x_ on the surface of the hybrid catalyst. However, the degradation product (TiO_2_) of the Ti_3_C_2_T_x_ is visible after OER (Fig. 10e).

For the 10% MXene, there is a larger amount of TiO_2_ detected after the OER when compared to the 1% MXene (Fig. 10e). This would cause the hybrid catalyst to lose stability when compared to the 1% MXene as TiO_2_ is an insulator. It is now evident from the XPS analysis that the lighter brown color of the 10% MXene film is due to the higher amount of TiO_2_ formed from the oxidation of the MXene as the initial ratio of Co:MXene is higher than the 1% MXene film. This higher amount of TiO_2_ in the film is evidently the reason behind the 10% MXene exhibiting poorer OER performances in terms of overall long-term activity.

The stability of the hybrid materials clearly depends on the amount of MXene in the composite, which is due to the oxidation of the MXene to TiO_2_. Therefore, less MXene in the hybrid is essential to prepare a catalyst with better long-term OER capabilities. Further studies on how the ratio of the MXene:metal oxides effect the OER, along with using other MXene materials, e.g., Nb_2_C, Ti_2_C, Mo_2_CT_x_, for the OER, will be undertaken by our group^28,29^.

This work has helped to establish a greater insight into the relationship between MXene and TMO components in active OER composite catalysts. There is no question that the addition of a small amount of Ti_3_C_2_T_x_ MXene as a conductive additive can have a positive effect on the initial water splitting capabilities of a Co(OH)_2_ electrocatalyst, demonstrated here via polarization and Tafel slopes. This research gives evidence of two principal effects of MXene integration on the performance. Firstly, the enhanced conductive matrix allows for the coordinated Co metals to reach a higher oxidation state at lower input potentials, demonstrated using operando Raman spectroscopy on electrocatalytically active electrodes. The high oxidation state Co(IV) stimulates reactivity at the catalyst surface with a lower overpotential compared to the pure catalyst. Secondly, this work suggests a mechanical enhancement in the bi-component catalyst evidenced by the robust observable film formed by the 1% MXene sample after electrochemical cycling, both in terms of cohesion of the film, and adhesion to the ITO surface.

With the inclusion of a MXene component, it is always necessary to consider stability for any practical application. Here, a “shut down/powering on” protocol is used by cyclic voltammetry around the expected onset potential for the cell, to simulate the response to intermittent electrolysis rather than constant current/potential experiments. As such, the tests were able to show superior response of 1% and 10% MXene composite catalysts to the “shut down/power up” input compared to the pure Co(OH)_2_ catalyst. Increasing the TMO:MXene ratio from 1% to 10% in the catalyst sees no improvement in the performance, in fact the performance over time is slightly decreased for the 10% MXene sample. After extensive cycling, MXene oxidation begins to become a factor as evidenced by ex situ XPS data, with a more negative effect experienced by the 10% MXene catalyst.

This work clearly demonstrates that the addition of small amounts of MXene material to metal oxides are advantageous for the preparation of OER catalysts. These TMO/MXene materials could provide alkaline water electrolysis systems with new, inexpensive, conductive and active OER catalysts needed to bring this technology to the forefront of sustainable energy for the masses.

## Methods

### Co(OH)_2_ synthesis

In a glass beaker, Co(NO_3_)_2_·6H_2_O (Merck), NaCl (Merck) and hexamethyltetramine (Merck) were dissolved in a mixture of water and ethanol (9:1% v/v) giving a final concentration of 10, 50 and 60 mM, respectively. The pink solution was then placed on a hot plate and heated to 90 °C for 1 h. During heating, the formation of green precipitate was observed. Solid particles were centrifuged and washed several times with water and ethanol.

### MXene synthesis

Into a PTFE bottle with vented lid, 9 M HCl (40 ml, Sigma) was added, followed by the addition of LiF powder (3.2 g, Sigma). The bottle was then placed in a mineral oil bath, stirring at 400 rpm using a magnetic PTFE stirrer bar for 10 min to fully dissolve the LiF. Ti_3_AlC_2_ MAX phase powder (2 g, Carbon-Ukraine ltd.) was then added in small additions to the vessel over a period of 30 min to avoid overheating of the solution. The temperature was then set to 35 °C for 24 h to obtain etched, multilayer Ti_3_C_2_T_x_ MXene (T_x_ = predominantly O, OH and F with some Cl). The contents of the bottle were transferred into 50 ml centrifuge tubes and diluted to a total of 160 ml with deionized water. The dispersion was then sedimented via centrifugation at 5000 rpm using a Thermo Scientific Heraeus Multifuge X1 for 5 min, discarding the supernatant and repeating several times, until the pH of the supernatant had reached at least 6. To delaminate the washed multilayer MXene, the dispersion was vortex mixed for 30 min. The dispersion was then centrifuged at 1500 rpm for 30 min to sediment any multilayer MXene or unreacted MAX phase. The supernatant containing delaminated MXene flakes was then collected. This supernatant was concentrated via centrifugation at 5000 rpm for 1 h. The sediments were redispersed in a minimum amount of deionized water to obtain the final MXene ink. To determine the concentration of the MXene ink, 100 μl was diluted with a 1:1 (v/v) ratio of deionized water and absolute ethanol (Fisher) before being filtered using a pre-weighed 0.25 μm pore nitrocellulose filter membrane (Millipore VSWP) and vacuum filtration flask. The vial and sides of the funnel were washed down with additional 1:1 deionized water and absolute ethanol. Once the filtration was complete the membrane and MXene filtride were dried overnight in a vacuum desiccator and then weighed to obtain the concentration, typically 30 mg/ml.

### TMO/MXene preparation

The hybrid TMO/MXene composites were prepared by preparing separate Co(OH)_2_ and MXene ink dispersions at a concentration of 10 mg/ml. The ink dispersions were made using 1:1 parts of H_2_O:EtOH and 8 µl of Nafion solution. Then the relevant percentage of the MXene ink dispersion was added to clean vial and then the Co(OH)_2_ ink dispersion was added to make up 100% volume.

### Electrochemical characterization

For the RDE experiments, 5 µl of the relevant ink dispersion was drop cast onto a Ti RDE and dried at room temperature. The ink dispersion was made up of 10 mg of the catalyst, 0.5 ml of H_2_O, 0.5 ml of EtOH and 8 µl of Nafion solution. The catalyst coated RDE was then inserted into a rotating ring set-up for measurements.

A standard three-electrode cell was utilized which consisted of the RDE with the catalyst as the working electrode, a Hg/HgO reference electrode and a carbon rod as the counter electrode. NaOH was used as the electrolyte. The linear sweep voltammetry (LSV) and Tafel slope measurements were carried out at a scan rate of 1 mV s^−1^. Cyclic voltammetry (CV) was performed at a scan rate of 40 mV s^−1^. The electrochemical impedance spectroscopy (EIS) measurements were carried out at a potential of 0 V vs. Hg/HgO (non-Faradaic region) from 100,000 to 0.12 Hz. The double layer capacitance measurements were performed by running multiple CVs with the scan rate ranging from 1 mV s^−1^ to 100 mV s^−1^. Then the potential at 0.9 V vs. RHE was plotted against scan rate and the slope was determined to get the double layer capacitance. The estimated electrochemical surface area was determined according to Jaramillo et al.^30^.

For the ITO based measurements, the catalysts were prepared by depositing a thin layer of active material (pure Co, 1% MXene, 10% MXene, and pure MXene) on the surface of a transparent indium tin oxide (ITO)/glass substrate using a sonication-assisted spray tool (USI Prism Ultracoat 300), held at 100 °C. A material flow rate of 0.5 ml/min was used and the mass loading was monitored externally using a Sartorious SE2 ultra-microbalance. The resulting catalyst was electrochemically cycled in a standard three-electrode setup with Hg/HgO reference and Pt counter electrodes, in 1 M NaOH electrolyte solution, and within the potential range 0–0.8 V vs. Hg/HgO at 5 mV s^−1^ scan rate using a BioLogic VMP 300 potentiostat for 400 CV cycles, or until the catalyst became unstable.

The potentials measured vs. Hg/HgO were mathematically converted to potential vs. reference hydrogen electrode (RHE) using the equation:

1 $$E_{{\rm{RHE}}} = E_{{\rm{Hg}}/{\rm{HgO}}} + 0.059 \cdot {\rm{pH}} + E^0_{{\rm{Hg}}/{\rm{HgO}}}$$

where $E^0_{{Hg}/{HgO}}$ is the standard $E_{{Hg}/{HgO}}$ electrode potential and is equal to 0.098 V at 25 °C.

### Materials characterization

Raman spectroscopy measurements were performed using a WITec alpha 300 R confocal Raman microscope. Raman analysis was performed directly on the ITO support using a 532 nm excitation source at 1 mW and a spectral grating with 1800 lines/mm.

Sample preparation for ex situ Raman experiments was identical to method described above for the active material deposited on ITO. For ex situ experiments, the catalyst was cycled in a standard three-electrode setup as before. After cycling for 500 times between 0–0.8 V vs. Hg/HgO, the catalyst was removed from the cell and rinsed with DI water then allowed to cool at RT before measuring.

Operando Raman experiments were performed directly in a Raman electrochemical flow cell (Redox.Me) with a sapphire window and using a Gamry Reference 600 potentiostat. A Pt counter and a Hg/HgO reference electrode was used in the operando cell. The various materials for analyzes were deposited on ITO supports.

### X-ray photoelectron spectroscopy

XPS measurements were obtained using an Omicron EA 125 Energy Analyzer with a monochromated Al K-alpha source at 1486.7 eV. High resolution core level component XPS scans were obtained with a pass energy of 20 eV, high magnification mode, entrance and exit slits of 6 mm and 3 mm, respectively, giving an overall source and instrument resolution of 0.6 eV.

### Scanning electron microscopy–energy dispersive X-ray spectroscopy (SEM_EDX)

OER electrodes were observed via scanning electron microscopy (SEM) (Zeiss GEMINI 0.1–30 keV, Carl Zeiss Microscopy, LLC, USA.) Energy dispersive X-ray (EDX) spectroscopy mapping was carried out using an Oxford Instruments 8 0 mm^2^ silicon drift detector.

### Transmission electron microscopy

The as-synthesized samples for TEM were prepared by dispersing the synthesized materials in DI water via ultrasonic bath sonication (Fisherbrand 11207 operated at 37 kHz). The dispersion was deposited on a lacey carbon 400 mesh Cu grid (01896-F, TED PELLA Inc.) by dropcast for observation, and allowed to dry in vacuum.

The samples were observed via field-emission (scanning) transmission electron microscopy ((S)TEM) (Titan, Thermo Fisher Scientific Inc.). The macrostructure of the nanosheets were analyzed using (S)TEM and an acceleration voltage of 300 kV was employed for both TEM and STEM imaging during the measurements.

Pre-OER samples were prepared by dispersing the synthesized materials in DI water via ultrasonic bath sonication (Fisherbrand 11207 operated at 37 kHz). Post-OER samples were cycled ten times between 0–0.7 V in a standard three-electrode cell with a Hg/HgO reference electrode and a carbon rod as the counter electrode. The catalyst was redispersed in DI water after cycling via ultrasonic bath sonication. The pre- and post-OER dispersions were deposited on lacey carbon 400 mesh Cu grids (01896-F, TED PELLA Inc.) by dropcast for observation, and allowed to dry in vacuum. The morphology and crystal structure of the samples were observed via (scanning) transmission electron microscopy ((S)TEM) imaging and selected area electron diffraction (SAED) (Titan 80–300 kV, Thermo Fisher Scientific Inc.). Elemental characterization of pre- and post-OER samples was carried out via energy dispersive x-ray spectroscopy (EDX) spectrum imaging (XFlash 6T-30 30 mm^2^ EDX detector, Bruker Corporation). An acceleration voltage of 300 kV was used for all imaging modalities.

### Supporting information

The Supporting Information is available free of charge at XXXX. The SI contains SEM and TEM images of the pure Co(OH)_2_ (Supplementary Fig. 1), STEM-EDX maps of the MXene (Supplementary Fig. 2), LSV curve of the bare Ti RDE support (Supplementary Fig. 3) and LSVs of the pure Co(OH)_2_, pure MXene, Ti support, 1% MXene prepared by physical mixing (PM) and 1% MXene prepared through a one-pot synthesis (H) (Supplementary Fig. 4), 1% MXene at 1.72 V vs. RHE showing peaks associated with Co_3_O_4_, Co(IV) and CoOOH (Supplementary Fig. 5) and electrochemical characterization of the Pure Co and 1% MXene samples (Supplementary Fig. 6).

### Supplementary information

Supplementary Information

## Data Availability

The data relating to the findings of this work is available from the corresponding author, subject to reasonable request.
