# Supplementary material for: Understanding the effect of MXene in a TMO/MXene hybrid catalyst for the oxygen evolution reaction
Source: NPJ 2D Mater Appl. 2023 Mar 10;7(1):15. doi: 10.1038/s41699-023-00377-1 (PMC11041736; doi:10.1038/s41699-023-00377-1)
Supplement: Supplementary file 1 — Supplementary Information [file 41699_2023_377_MOESM1_ESM.pdf]

Supporting Information

Understanding the Effect of MXene in a TMO/MXene Hybrid Catalyst for the Oxygen Evolution Reaction

Daire Tyndall[a,b], Lee Gannon[a,c], Lucia Hughes[a,b], Julian Carolan [a,b], Sergio Pinilla[a,b], Sonia Jaśkaniec[a,b], Dahnan Spurling[a,b], Oskar Ronan[a,b], Cormac McGuinness[a,c], Niall McEvoy[a,b], Valeria Nicolosi[a,b,d]* and Michelle P. Browne[a,b,e]*

[a] Centre for Research on Adaptive Nanostructures and Nanodevices (CRANN), Advanced Materials and BioEngineering Research (AMBER) Centre, Trinity College Dublin, Ireland.
[b] School of Chemistry, Trinity College Dublin, Ireland.
[c] School of Physics, Trinity College Dublin, Ireland.
[d] I-Form Research Center, Trinity College Dublin, Dublin, Ireland.
[e] Helmholtz-Zentrum Berlin für Materialien und Energie, 14109, Berlin, Germany.

*michelle.browne@helmholtz-berlin.de & nicolov@tcd.ie

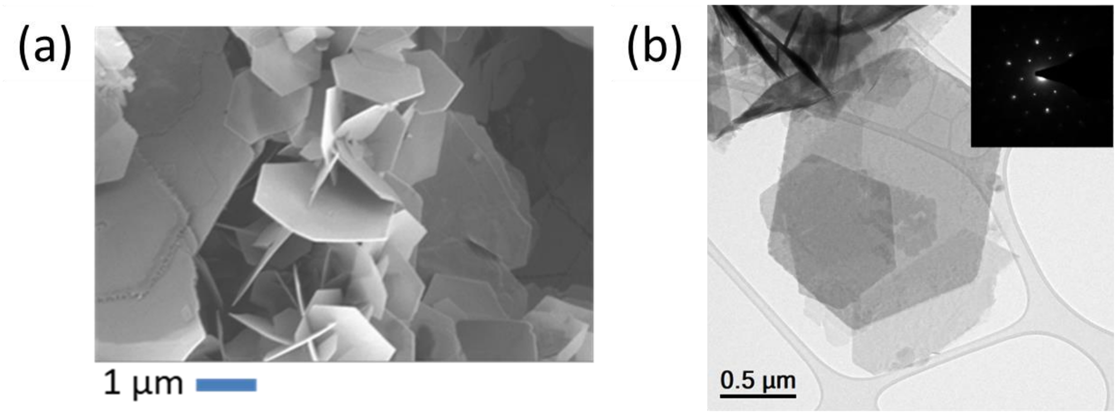

Supplementary Figure 1. Microscopy. a. InLens SEM image of $Co(OH)_2$ nanosheets. **b.** Bright Field TEM images of the Pure Co nanosheets viewed along [0001] axis. Inset Selected Area Electron Diffraction (SAED) pattern confirming hexagonal crystal structure of Pure Co nanosheets with $(01\bar{1}0)$ and $(10\bar{1}0)$ diffraction spots clearly present.

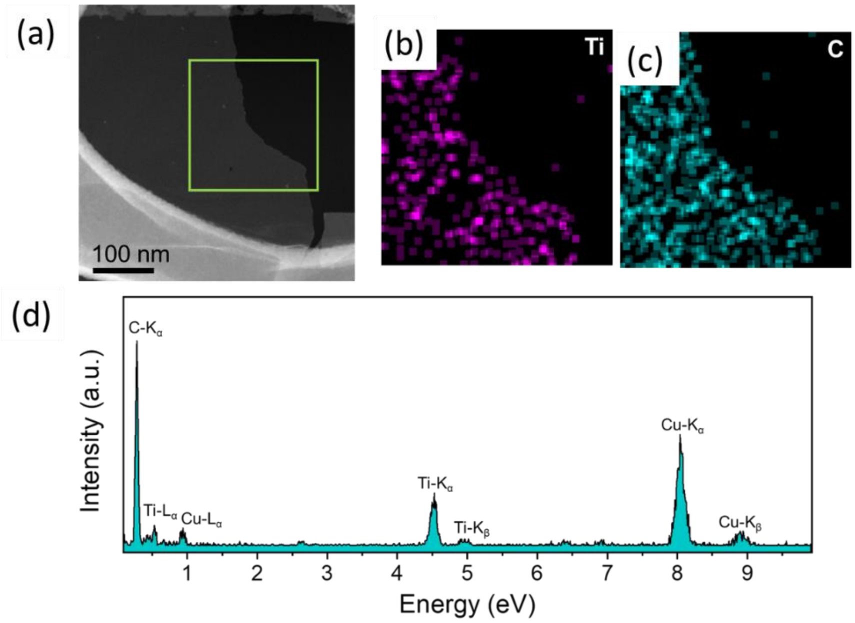

Supplementary Figure. 2 STEM of pure MXene. **a.** Scanning transmission electron microscopy image of the MXene. **b.** Energy dispersive X-Ray spectroscopy map of the Ti Kα corresponding to Figure S2A. **C.** Energy dispersive X-Ray spectroscopy map of the C Kα corresponding to Figure S2A and **D.** Corresponding EDX spectrum (Copper signal due to the copper TEM grid).

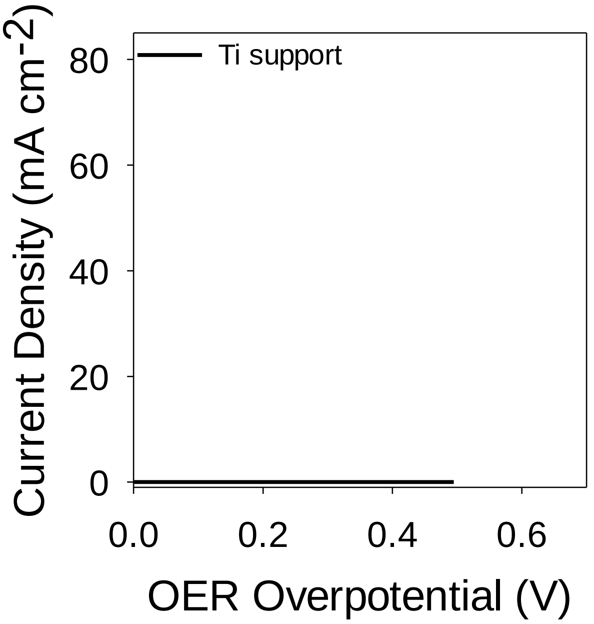

Supplementary Figure 3. OER TI support. LSV curve of the Ti support at a scan rate of 1 mV dec^{-1} at a rotation speed of 1600 rpm

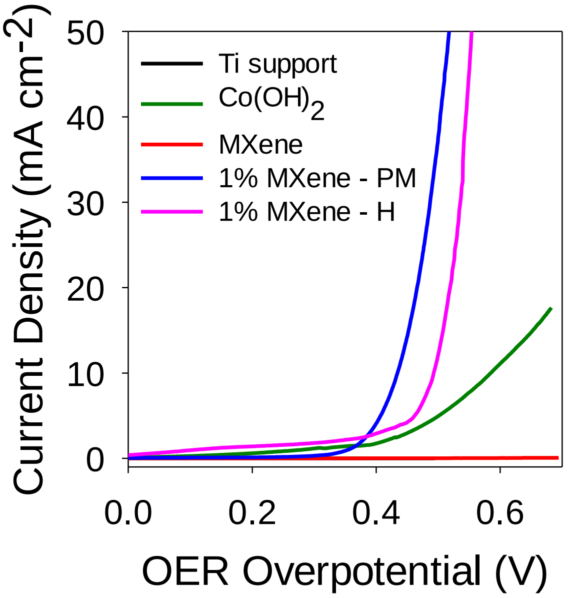

Supplementary Figure 4. PM vs. H OER. LSVs of the pure $Co(OH)_2$, pure MXene, Ti support, 1 % MXene prepared by physical mixing (PM) and 1% MXene prepared through a one-pot synthesis (H).

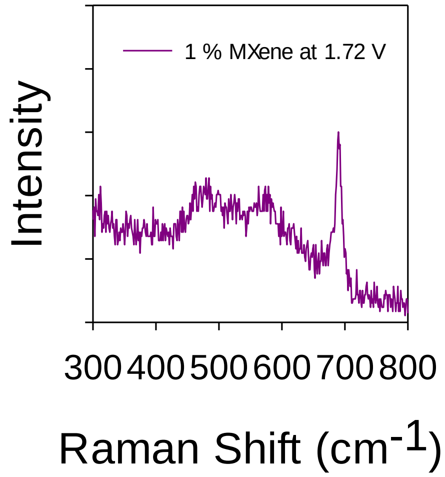

Supplementary Figure 5. 1 % MXene operando Raman. 1 % MXene at 1.72 V vs. RHE showing peaks associated with Co_3O_4, Co(IV) and CoOOH.

Supplementary note 1: Electrochemical testing approach to mimic 'the shut down/powering on" function of electrolysers'

As this technology will be implemented along with intermittent sources of energy, such as solar or wind, the performance of the cell before and after 'shut-down' is of critical importance.[1-2] As, if performance is lost when the cell is 'turned back on' this is a rise for concern. Hence, this work utilizes multi-cyclic voltammetry (CV) testing over a time to periodically 'shut-down' the cell and 'power on' again while tracking the changes within the active material. Other reports utilize accelerated stress tests (AST) which apply alternating high current density (mimics periods of operation) and low currents density (mimics no operation).[3] Multi-cycling the potential in and out of the OER also mimics periods of operation (power on) and no operation (shut down).

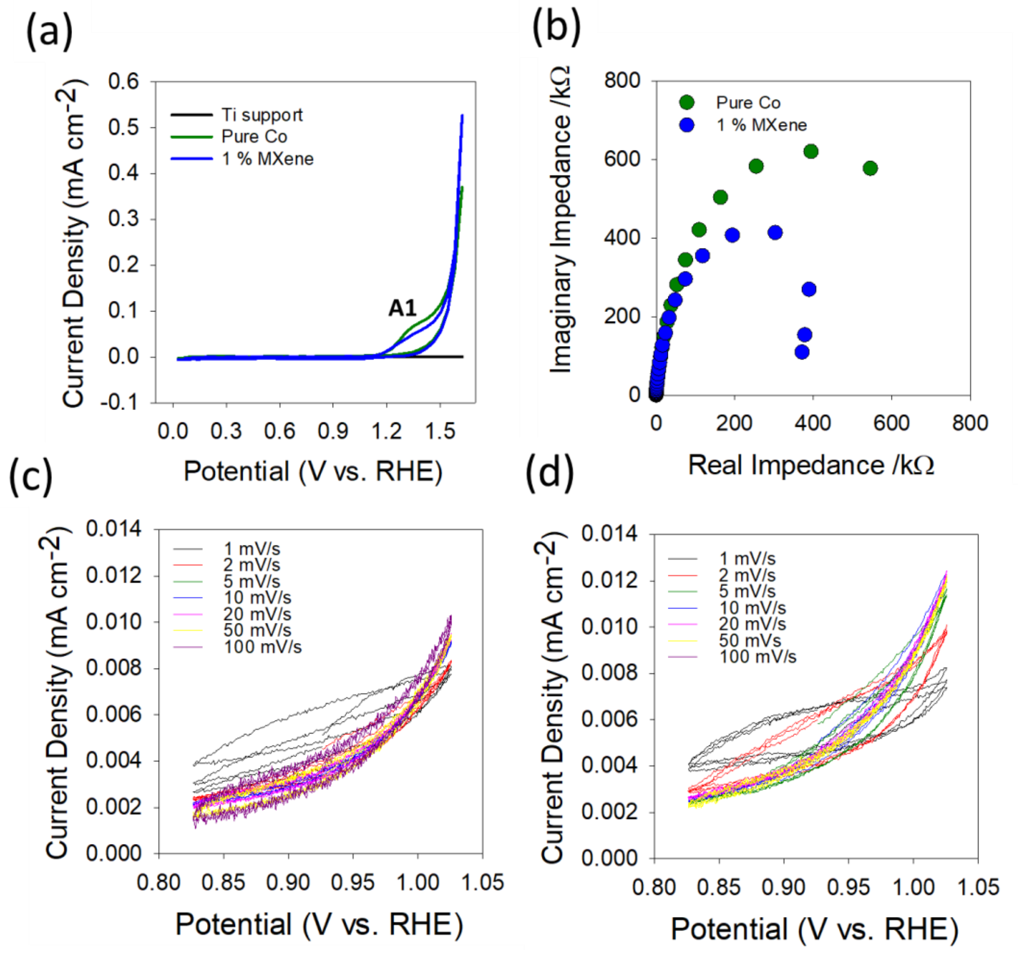

Supplementary Figure 6. Electrochemical characterisation. Electrochemical characterisation of the Pure Co and 1% MXene on a Ti support **a.** Cyclic Voltammetry at a scan rate of 40 mV/s **b.** Electrochemical Impedance Spectroscopy **c.** Multiple CVs in over a scan rate range of 1 – 100 mV/s for the pure Co and **d**. Multiple CVs in over a scan rate range of 1 – 100 mV/s for the 1 % MXene.

Supplementary note 2. The typical cyclic voltammograms for the pure Co and 1% MXene can be observed in Figure S6A. Both materials exhibit one broad redox peak (A1) in the potential window of ~1.15 - 1.35 V vs. RHE prior to the OER. Based on the Raman spectroscopy in this study and literature reports, this redox peak can be assigned to a Co(II)/Co(III) to a Co(III) re-arrangement transition.[4] As reported by Lyons and co-workers, the Co redox transitions can be extremely complicated in this potential region and include the oxidation/re-arrangement of Co(II)/Co(III) to Co(III) species such as which include Co_3O_4, CoOOH and Co_2O_3. There is no evidence of the Co(III) to Co(IV) redox transition for the pure Co and 1 % MXene in Figure S6 prior to OER.

The electrochemical impedance spectroscopy suggests that the 1% MXene should have lower charge transfer resistances compared with the pure Co as the semi-circle for the 1% MXene has a small radius from the Nyquist plot in Figure S6B.

From Figure 6C-D, the capacitance of each material was calculated using the multi-CV method at various scan rates and by extracting the current density at a particular potential. Interestingly for the two materials, the current density decreases on the positive sweeps at the potential of 0.9 V vs. RHE at the scan rates of 1 -5 mV/s, therefore the capacitance for each material was calculated using the

scan rates of 10 – 100 mV/s. The capacitance of the pure Co is 0.0042 mF cm^{-2} and for the 1 % MXene the capacitance is 0.0057 mF cm^{-2}. Hence, the 1% MXene materials has a slightly larger capacitance but not large enough to explain the increase in the OER performance. These capacitance values correspond to estimated electrochemical surface area (ECSA) values of 0.105 cm^2 for the pure Co and 0.1425 cm^2 for the 1 % MXene.

References

1. Hug, W.; Bussmann, H.; Brinner, A., Intermittent operation and operation modeling of an alkaline electrolyzer. *International Journal of Hydrogen Energy* **1993,** *18* (12), 973-977.
2. Furfari, S.; Clerici, A., Green hydrogen: the crucial performance of electrolysers fed by variable and intermittent renewable electricity. *The European Physical Journal Plus* **2021,** *136* (5), 509.
3. Weiß, A.; Siebel, A.; Bernt, M.; Shen, T. H.; Tileli, V.; Gasteiger, H. A., Impact of Intermittent Operation on Lifetime and Performance of a PEM Water Electrolyzer. *Journal of The Electrochemical Society* **2019,** *166* (8), F487.
4. Lyons, M.E.G, Brandon, M.P., The Oxygen Evolution Reaction on Passive Oxide Covered Transition Metal Electrodes in Alkaline Solution Part II – Cobalt, *International Journal of Electrochemical Sciences* **2008**, 3 1425 – 1462.
